# Next‐generation sequencing reveals the presence of *DDX41* mutations in acute lymphoblastic leukemia and aplastic anemia

**DOI:** 10.1002/jha2.256

**Published:** 2021-06-27

**Authors:** Yang Zhang, Fang Wang, Xue Chen, Hong Liu, Xiaoliang Wang, Jiaqi Chen, Panxiang Cao, Xiaoli Ma, Hongxing Liu

**Affiliations:** ^1^ Divison of Laboratory Medicine Hebei Yanda Lu Daopei Hospital Langfang China; ^2^ Divison of Laboratory Medicine Beijing Lu Daopei Hospital Beijing China; ^3^ Beijing Lu Daopei Institute of Hematology Beijing China

**Keywords:** acute lymphoblastic leukemia, aplastic anemia, DDX41 mutations, genetic predisposition

## Abstract

Limited studies have been described DEAD‐box helicase 41 (*DDX41)* mutations in hematological diseases other than myeloid neoplasms. In this study, *DDX41* mutations were identified in 0.8% of myeloid neoplasms, 0.9% of acute lymphoblastic leukemia (ALL), and 1.0% of aplastic anemia (AA). A total of 15 causal *DDX41* variants in 14 patients were detected; seven of which have not been reported previously. In myeloid neoplasms, the median age of patients with germline missense was lower than that of germline nonsense mutations. In ALL, the characteristics of *DDX41* mutation were distinct. This study first reported *DDX41* mutations in ALL and AA, expanding its mutation and phenotypic spectrum.

## 1 INTRODUCTION

Myeloid neoplasms with germline DEAD‐box helicase 41 (*DDX41)* mutation have been included in the 2016 revised World Health Organization (WHO) myeloid neoplasms classification as a new diagnostic category [1]. *DDX41* mutation has been reported mainly in about 2%–5% of myeloid neoplasms [2, 3, 4, 5, 6], a few in chronic myeloid leukemia, lymphomas, multiple myeloma, sarcoidosis, and blastic plasmacytoid dendritic cell neoplasm [2, 7]. However, limited studies are describing the mutation profile of *DDX41* other than myeloid neoplasms in hematological disease. We analyzed the prevalence and characteristics of *DDX41* mutations in an unselected cohort of patients with hematological disorders to advance our understanding of this gene.

## 2 METHODS

### 2.1 Patients

From February 2017 to May 2020, bone marrow and peripheral blood samples from 1753 patients who were admitted at Hebei Yanda Lu Daopei Hospital were collected, including 720 subjects with acute myeloid leukemia (AML), 91 with myelodysplastic syndromes (MDS), 41 with myeloproliferative neoplasms (MPN), 16 with MDS/MPN, 585 with B‐cell acute lymphoblastic leukemia (B‐ALL), 164 with T‐cell ALL (T‐ALL), 42 with mixed phenotype acute leukemia, and 94 with aplastic anemia (AA). The diagnosis criteria were referred to the 2016 World Health Organization classification of myeloid neoplasms and acute leukemia [1].

### 2.2 Gene sequencing and mutation analysis

Mutational hotspots or whole coding regions of 86 genes (Table S1) known to be frequently mutated in hematologic malignancies (HMs) were sequenced using a targeted amplicon‐based high‐throughput sequencing protocol, as we previously reported [8]. *DDX41* genes were tested for the whole coding region in this multigene panel. *DDX41* germline variants were classified for pathogenicity according to the American College of Medical Genetics and Genomics guidelines [9], and acquisition of a somatic *DDX41* mutation was also considered as a firm criterion for causality. The fingernail specimens or blood samples in complete remission (CR) of acute leukemia were taken as a control to verify the possible germline origin.

### 2.3 Follow‐up

The end of the follow‐up period was December 30, 2020. CR was defined as morphologic CR. Overall survival (OS) is measured from the date of diagnosis to the date of death or the date of the last follow‐up; relapse‐free survival (RFS) is measured from the date of achievement of CR until the date of relapse, death, or to the date of the last follow‐up.

### 2.4 Statistical analysis

Statistical analysis was performed using the SPSS 22.0 software, and chi‐square or Fisher's exact tests calculated the significance between categorical data. A *p*‐value of <0.05 was considered statistically significant.

## 3 RESULTS

### 3.1 The characteristics of *DDX41* mutations

**FIGURE 1:**
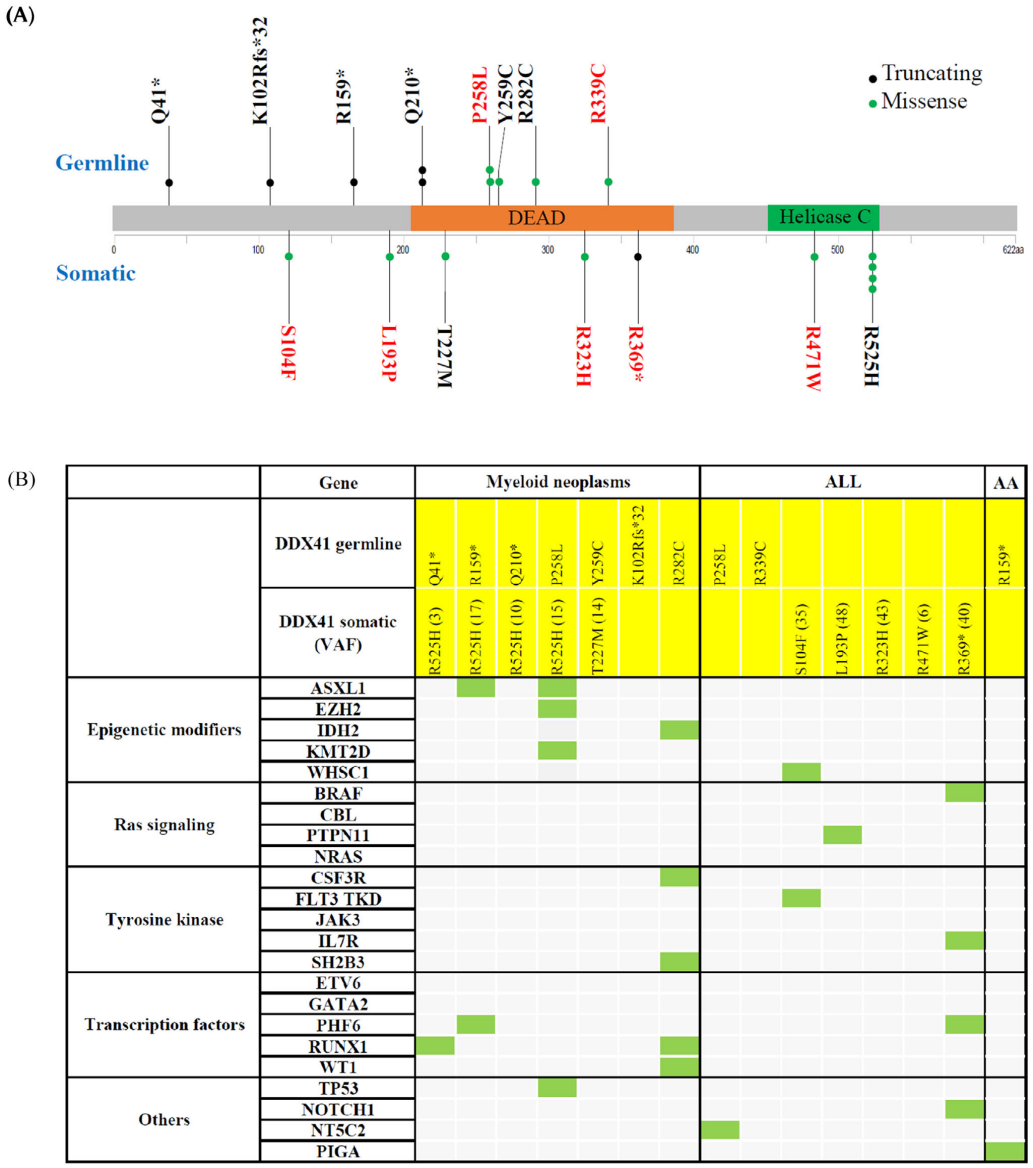
(A) Schematic diagram of DDX41 mutations in hematological disease. Germline mutations are in the upper part and somatic mutations in the lower part. The number of dots represents the case number of the mutation observed in this cohort. The color of the dots indicates the mutation type; black is a truncating, and green is a missense mutation. The variants in red color were detected in ALL. (B) The DDX41 and concomitant mutations in each patient with myeloid neoplasms, ALL, and AA. Row in the graph represents individual mutated genes, and columns represent individual patients

A total of 29 *DDX41* variants were identified, including 22 germline variants and seven somatic variants. The germline variants were classified as causal (*n* = 8) and uncertain significance (*n* = 14) (Table S2); the latter were excluded in this assay. Seven causal variants (K102Rfs*32, S104F, L193P, Q210*, R282C, R323H, R471W) have not been reported previously. All of the germline variants were located on or upstream of the DEAD domain. Somatic *DDX41* variants occurred throughout the whole coding region in ALL, but in the myeloid neoplasm, 80% of them were hotspot R525H (Figure 1A).

### 3.2 *DDX41* mutations in myeloid neoplasm

*DDX41* mutations were identified in 0.8% of patients (six AML, one chronic myelomonocytic leukemia [CMML]) with myeloid neoplasm. The median age was 59 years (range, 28–78 years), and 57% were male. None of the patients had del 5/5q. In six patients with AML, four cases (66.7%) were arising from previous MDS; one case had a history of thrombocytopenia for 2 years before diagnosed with AML. Two patients had a family history of anemia and leukemia, respectively. Germline nonsense and missense mutation were respectively detected in three patients with AML, five of which acquired a somatic *DDX41* mutation (4 R525H, 1 T227M), the remaining one concomitant with five somatic gene mutations (CSF3R, IDH2, RUNX1, SH2B3, WT1) (Figure 1B). The median age of patients with germline missense was lower than that with germline nonsense mutations (37 years vs. 70 years, *p = 0.100*).

### 3.3 DDX41 mutations in ALL and AA

*DDX41* mutations were identified in 0.9% of the ALL cohort (1.0% of B‐ALL (*n* = 6), 0.6% of T‐ALL (*n* = 1)). The median age was 9 years (range, 4–27 years), and 56% were male. None of the patients had a family history of hematological malignancy and del 5/5q. Somatic (four missenses, one nonsense) and germline *DDX41* mutations (two missenses) were respectively detected in five and two patients, which appeared to be mutually exclusive because no biallelic mutations were detected (Figure 1B).

One case of AA in this cohort was observed with *DDX41* mutations. He was diagnosed with AA at eight years old and treated with cyclosporin A for five years. Then he developed a drop in blood cell count, abdominal pain, and dark‐colored urine. Flow cytometry showed that the paroxysmal nocturnal hemoglobinuria (PNH) clone size in red blood cells was 77.2%, and in granulocyte was 96.7%. *DDX41* germline mutation (R159*, variant allele frequency [VAF] 50%) and *PIGA* mutations (c.1188+1G > A, VAF 36%; L110Cfs*13, VAF 14%) were identified. The diagnosis was modified to AA‐PNH syndrome. His parents are young (36 years) and healthy, without a family history of tumors.

### 3.4 Prognosis of patients with *DDX41* mutations

Among six patients with AML, two received treatment, and the rest were abandoned after diagnosis. One relapsed after hematopoietic stem cell transplantation (HSCT) and received the second HSCT; the other one received 10 courses of chemotherapy and showed continuous no remission, then abandoned. Among five patients with ALL carried somatic *DDX41* mutation, three relapsed and the median RFS was 21 months; four received HSCT (Table 1). Only one of ALL patients with germline *DDX41* mutation was available to follow‐up, which was continued to be minimal residual disease positive and then received HSCT. The patient with AA‐PNH syndrome eventually received HSCT due to persistent, recurrent abdominal pain and dark‐colored urine, and the need for platelet and red blood cell transfusion. At the time of this writing, the disease‐free survival was 9 months after transplantation.

**TABLE 1:** Clinical and genetic features of patients with DDX41 mutations

							DDX41 mutation	
Number	Age (years)	Gender	Diagnosis	Cytogenetics	Fusion gene	Familial history	Germline	Somatic (VAF)	Treatment	Status	Time of OS (m)	Time of DFS (m)
1	70	M	AML‐MRC	normal karyotype	Negative	Anemia	R159*	R525H (17)	Palliative treatment	Deceased	33	—
2	78	F	Primary AML	Normal karyotype	Negative	No	Q210*	R525H (10)	Palliative treatment	Loss to follow‐up	—	—
3	59	M	AML‐MRC	Normal karyotype	Negative	No	Y259C	T227M (14)	Palliative treatment	Alive	9	—
4	68	M	AML‐MRC	Normal karyotype	Negative	No	Q41*	R525H (3)	Palliative treatment	Loss to follow‐up	—	—
5	37	M	AML‐MRC	46,XY,add(9)(p22)[20]	Negative	No	P258L	R525H (15)	Chemotherapy, HSCT	Deceased	74	35
6	28	F	primary AML	46,XX,‐12,der(22)t(12;22)(q13;p11.2) +mar1[11]/46,XX[12]	Negative	Leukemia	R282C		Chemotherapy	Deceased	31	—
7	56	F	CMML	46,XX,t(11;19)(q23;p13.1)[2]/46,XX[37]	MLL‐ELL	No	K102Rfs*3		Chemotherapy	Alive	11	—
8	9	F	B‐ALL	48,XY,+del(1)(p12p21),‐4,+8,add(9)(p24),(der21)t(1;21)(p21;q11.2),+22[20]	Negative	No	P258L		Not available	Loss to follow‐up	—	—
9	6	F	B‐ALL	Normal karyotype	BCR‐ABL1	No	R339C		Chemotherapy, HSCT	Alive	19	—
10	7	M	B‐ALL	Normal karyotype	TEL‐AML1	No		S104F (35)	Chemotherapy, HSCT	Alive	71	28
11	9	M	B‐ALL	46,XY,t(17;19)(q22;p13.3)[4]/46,XY[7]	E2A‐HLF	No		L193P (48)	Chemotherapy, CAR‐T, HSCT	Alive	17	—
12	4	F	B‐ALL	Normal karyotype	Negative	No		R323H (43)	Chemotherapy	Alive	25	—
13	16	M	B‐ALL	Normal karyotype	E2A‐PBX1	No		R471W (6)	Chemotherapy, CAR‐T, HSCT	Deceased	25	12
14	27	M	T‐ALL	46,XY,der(1)t(1;1)(p36.1;q25),del(4)(q23),del(6)(p23),inv(7)(q21q22),der(11)t(X;11)(q13;p15),del(13)(q12q14)[14]//46,XX[8]	Negative	No		R369* (40)	Chemotherapy, HSCT	Alive	54	21

Abbreviations: AML‐MRC, AML with myelodysplasia‐related changes; DFS, disease‐free survival; F, female; M, male; m, months; OS, overall survival; VAF, variant allele frequency.

## 4 DISCUSSION

Previous studies have reported that *DDX41* mutations were identified in 3% of families with suspected inherited HMs [2]. in 3.1% of Han Chinese patients with myeloid neoplasms [4], in 5.5% of Thai patients with myeloid neoplasms [3]. In this cohort, *DDX41* mutations were found only in 0.8% of patients with myeloid neoplasms. It might be related to the composed of mainly primary AML patients in this cohort. The median age of this cohort is 28 years, which is lower than the mean age of onset (62 years) of HMs that have been reported [2]. However, our results also support that most AML cases who were carrying *DDX41* mutations have an MDS history and are older.

There are a few reports of *DDX41* mutations in lymphoid malignancies; it was speculated that dysregulation of the innate immune response might be linked to lymphoid malignancy [2]. However, it has not been reported in ALL. We identified *DDX41* mutations in 0.9% of patients with ALL, and the characterize was distinct from myeloid tumors; with a young age of onset, somatic and germline *DDX41* variants appeared to be mutually exclusive, the majority (80.0%) of the somatic variants were located on or upstream of the DEAD domain. It suggested that the pathogenesis of *DDX41* mutations in ALL may differ from myeloid tumors, which merits further study.

Compared with other myeloid neoplasm predisposition syndromes, patients with germline *DDX41* mutations are older at the time of presentation [10, 11]. Loss of function (LOF) germline *DDX41* mutations is by far most prevalent. However, the age of onset at different mutation sites may differ, as suggested by some reports. Missense germline mutations in the helicase C domain of *DDX41* have been observed to cause an earlier onset disease than those with LOF mutations [2]. Consistent with previous reports, we also observed that patients with a missense germline *DDX41* mutation have a lower onset age than nonsense germline mutation carriers (37 years vs. 70 years). The notable difference from the previous is that the missense mutation we observed is located at the N‐terminal DEAD‐box domain. It indicates that patients with *DDX41* missense mutations have an early age of onset, which is irrelevant to the domain where it occurs.

The p. D140fs or p.M1I variants were previously reported as the most common germline *DDX41* mutations in the Caucasian population [2, 5]. However, it is rare in East and Southeast Asian populations [3, 4], neither was detected in this cohort. It is indicating that distinct ethnically associated with recurrent germline mutations. Approximately 50% of patients with germline *DDX41* mutations also harbor somatic mutations in the other allele as a double hit event during progression to MDS or AML [5, 12] with the missense mutation p.R525H being most common [13, 14]. However, we and others have observed that individuals with single germline *DDX41* mutations also progressed to hematological tumors [15]. In this cohort, we found one patient with AML concomitant with CSF3R, IDH2, RUNX1, and WT1 mutations, one patient with CMML concurrent with the MLL‐ELL fusion gene. We speculate that these mutations or fusion genes may interact with germline *DDX41* variants in the development of myeloid neoplasms.

In conclusion, we first reported the *DDX41* mutations in ALL and AA, which have distinct characteristics, thereby expanding the mutation and phenotypic spectrum of *DDX41* mutations. Moreover, the genotype‐phenotype correlations regarding *DDX41* mutations should be clarified more specifically in the future.

## CONFLICT OF INTEREST

The authors declare no conflict of interest.

## AUTHOR CONTRIBUTIONS

Hongxing Liu designed the research and critically revised the paper. Yang Zhang performed the research and wrote the paper. Fang Wang and Xue Chen supervised clinical and experimental findings. Hong Liu, Xiaoliang Wang, Jiaqi Chen, and Xiaoli Ma analyzed the data. Panxiang Cao performed bioinformatics analysis. All the authors read and approved the final version of the manuscript.

## ETHICS APPROVAL

This study was approved by the Institutional Review Board and Ethical Committee of the Hebei Yanda Lu Daopei Hospital.

## PATIENT CONSENT STATEMENT

Written informed consent was obtained from all the patients.

## Supporting information

SUPPORTING INFORMATION

SUPPORTING INFORMATION

## Data Availability

The data that support the findings of this study are available upon request from the corresponding author. The data are not publicly available due to privacy or ethical restrictions.

## References

[1] Arber DA , Orazi A , Hasserjian R , Thiele J , Borowitz MJ , Le Beau MM , et al. The 2016 revision to the World Health Organization classification of myeloid neoplasms and acute leukemia. Blood. 2016;127(20):2391–405. 27069254 10.1182/blood-2016-03-643544

[2] Lewinsohn M , Brown AL , Weinel LM , Phung C , Rafidi G , Lee MK , et al. Novel germline DDX41 mutations define families with a lower age of MDS/AML onset and lymphoid malignancies. Blood. 2016;127(8):1017–23. 26712909 10.1182/blood-2015-10-676098 PMC4968341

[3] Polprasert C , Takeda J , Niparuck P , Rattanathammethee T , Pirunsarn A , Suksusut A , et al. Novel DDX41 variants in Thai patients with myeloid neoplasms. Int J Hematol. 2020;111(2):241–6. 31713024 10.1007/s12185-019-02770-3

[4] Qu S , Li B , Qin T , Xu Z , Pan L , Hu N , et al. Molecular and clinical features of myeloid neoplasms with somatic DDX41 mutations. Brit J Haematol. 2020;192(6):1006–10. 32307695 10.1111/bjh.16668 PMC9205684

[5] Polprasert C , Schulze I , Sekeres MA , Makishima H , Przychodzen B , Hosono N , et al. Inherited and somatic defects in DDX41 in myeloid neoplasms. Cancer Cell. 2015;27(5):658–70. 25920683 10.1016/j.ccell.2015.03.017 PMC8713504

[6] Sébert M , Passet M , Raimbault A , Rahmé R , Raffoux E , Sicre De Fontbrune F , et al. Germline DDX41 mutations define a significant entity within adult MDS/AML patients. Blood. 2019;134(17):1441–4. 31484648 10.1182/blood.2019000909

[7] Diness BR , Risom L , Frandsen TL , Hansen B , Andersen MK , Schmiegelow K , et al. Putative new childhood leukemia cancer predisposition syndrome caused by germline bi‐allelic missense mutations in DDX41. Genes Chromosomes Cancer. 2018;57(12):670–4. 30144193 10.1002/gcc.22680

[8] Zhang Y , Wang F , Chen X , Zhang Yu , Wang M , Liu H , et al. CSF3R mutations are frequently associated with abnormalities of RUNX1, CBFB, CEBPA, and NPM1 genes in acute myeloid leukemia. Cancer. 2018;124(16):3329–38. 29932212 10.1002/cncr.31586

[9] Richards S , Aziz N , Bale S , Bick D , Das S , Gastier‐Foster J , et al. Standards and guidelines for the interpretation of sequence variants: a joint consensus recommendation of the American College of Medical Genetics and Genomics and the Association for Molecular Pathology. Genet Med. 2015;17(5):405–23. 25741868 10.1038/gim.2015.30 PMC4544753

[10] Weinberg OK , Kuo F , Calvo KR . Germline predisposition to hematolymphoid neoplasia. Am J Clin Pathol. 2019;152(3):258–76. 31309983 10.1093/ajcp/aqz067 PMC7179513

[11] Bannon S , Dinardo C . Hereditary predispositions to myelodysplastic syndrome. Int J Mol Sci. 2016;17(6):838. 10.3390/ijms17060838 PMC4926372 27248996

[12] Maciejewski JP , Padgett RA , Brown AL , Müller‐Tidow C . DDX41‐related myeloid neoplasia. Semin Hematol. 2017;54(2):94–7. 28637623 10.1053/j.seminhematol.2017.04.007 PMC8190973

[13] Kadono M , Kanai A , Nagamachi A , Shinriki S , Kawata J , Iwato K , et al. Biological implications of somatic DDX41 p.R525H mutation in acute myeloid leukemia. Exp Hematol. 2016;44(8):745–54.e4. 27174803 10.1016/j.exphem.2016.04.017

[14] Quesada AE , Routbort MJ , DiNardo CD , Bueso‐Ramos CE , Kanagal‐Shamanna R , Khoury JD , et al. DDX41 mutations in myeloid neoplasms are associated with male gender, TP53 mutations and high‐risk disease. Am J Hematol. 2019;94(7):757–66. 30963592 10.1002/ajh.25486

[15] Berger G , Van Den Berg E , Sikkema‐Raddatz B , Abbott KM , Sinke RJ , Bungener LB , et al. Re‐emergence of acute myeloid leukemia in donor cells following allogeneic transplantation in a family with a germline DDX41 mutation. Leukemia. 2017;31(2):520–2. 27795557 10.1038/leu.2016.310

